# AlGaN/GaN High Electron Mobility Transistor-Based Biosensor for the Detection of C-Reactive Protein

**DOI:** 10.3390/s150818416

**Published:** 2015-07-28

**Authors:** Hee Ho Lee, Myunghan Bae, Sung-Hyun Jo, Jang-Kyoo Shin, Dong Hyeok Son, Chul-Ho Won, Hyun-Min Jeong, Jung-Hee Lee, Shin-Won Kang

**Affiliations:** School of Electronics Engineering, Kyunpook National University, 80 Daehakro, Buk-gu, Daegu 702-701, Korea; E-Mails: heeholee@ee.knu.ac.kr (H.H.L.); mhbae@ee.knu.ac.kr (M.B.); shjo@ee.knu.ac.kr (S.-H.J.); dhson@ee.knu.ac.kr (D.H.S.); chwon@ee.knu.ac.kr (C.-H.W.); hmjeong@ee.knu.ac.kr (H.-M.J.); jlee@ee.knu.ac.kr (J.-H.L.); swkang@knu.ac.kr (S.-W.K.)

**Keywords:** AlGaN/GaN, HEMT, CRP, biosensor, null-balancing circuit

## Abstract

In this paper, we propose an AlGaN/GaN high electron mobility transistor (HEMT)-based biosensor for the detection of C-reactive protein (CRP) using a null-balancing circuit. A null-balancing circuit was used to measure the output voltage of the sensor directly. The output voltage of the proposed biosensor was varied by antigen-antibody interactions on the gate surface due to CRP charges. The AlGaN/GaN HFET-based biosensor with null-balancing circuit applied shows that CRP can be detected in a wide range of concentrations, varying from 10 ng/mL to 1000 ng/mL. X-ray photoelectron spectroscopy was carried out to verify the immobilization of self-assembled monolayer with Au on the gated region.

## 1. Introduction

C-reactive protein (CRP) is a ring-shaped pentameric protein found in blood plasma, whose initial role as a pattern recognition molecule may have been to defend against bacterial infection. It is protective against a variety of bacterial infections [1]. In humans, CRP rises within two hours of the onset of inflammation, up to 50,000-fold, and peaks at 48 h. Its half-life of 18 h is constant, and therefore its level is determined by the rate of production and, hence, the severity of the precipitating cause [2]. Therefore, measuring and charting CRP concentration can prove useful in determining disease progress or the effectiveness of treatments [3].

A variety of chemical and optical methods are available for the detection of CRP, including enzyme-linked immunosorbent assay (ELISA), visual agglutination, and immunoturbidimetry [4,5,6]. However, these methods require the use of various fluorescent materials and large-size equipment. These problems can be overcome by electrical sensors, such as field effect transistor-type biosensors [7,8].

The AlGaN/GaN-based heterostructure shows unique electronic properties, such as piezoelectric and spontaneous polarization, which lead to high mobility and high density of two-dimensional electron gas (2DEG) in the interface of the heterostructure [9,10]. The conducting channel of the AlGaN/GaN high electron mobility transistor (HEMT) is very close to the surface and extremely sensitive under applied stress, which should enhance detection sensitivity [11,12]. The application of some external change in surface conditions, such as the binding of biomolecules to the gate region of the device, changes the piezoelectric-induced carrier density in the channel of the HEMT, which in turn alters the drain current [13]. Therefore, the HEMT-based biosensor provides high sensitivity to immobilized biomolecules. Moreover, the HEMT can be combined with a null-balancing circuit for measuring the surface potential of a field-effect transistor (FET)-type biosensor. The advantages of the null-balancing circuit include direct measurement of the sensor output signal without a bulky semiconductor parameter analyzer, and suitability of the device configuration for signal processing [14,15].

In this study, we investigated the feasibility of detecting a CRP from human plasma using an AlGaN/GaN HEMT-based biosensor with a null-balancing circuit. The null-balancing circuit is used to measure the change of the gate potential. The electrical characteristics of the biosensor were measured after immobilization of self-assembled monolayer (SAM), CRP-antibody, and CRP. We then monitored the output voltage of the biosensor with null-balancing circuit applied to detect changes in the potential measured at the gate surface; these changes were produced by the immune reaction of the CRP-antibody to CRP. Troponin T protein was used to evaluate the selectivity property of the AlGaN/GaN HEMT-based biosensor for CRP detection. The AlGaN/GaN HEMT-based biosensor with null-balancing circuit detected CRP at a concentration as low as 10 ng/mL in phosphate buffered saline (PBS) solution. We verified SAM immobilization on the Au surface using X-ray photoelectron spectroscopy (XPS) measurements.

## 2. Experimental Details

### 2.1. Fabrication of the AlGaN/GaN HEMT-Based Biosensor

**Figure 1:**
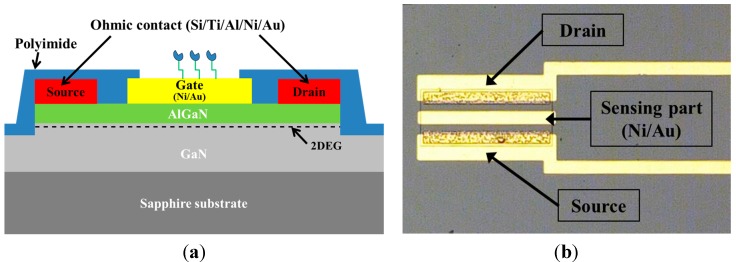
(**a**) Schematic of the AlGaN/GaN HEMT-based biosensor; (**b**) Photograph of the fabricated AlGaN/GaN HEMT-based biosensor.

Figure 1a shows the schematic configuration of the AlGaN/GaN HEMT-based biosensor. The AlGaN/GaN heterostructure was grown using metal-organic chemical vapor deposition (MOCVD) on a sapphire substrate, which consists of a 2-μm-thick resistive GaN layer, an 80-nm-thick undoped GaN layer, and a 25-nm-thick Al_0.3_Ga_0.7_N layer with 30% Al composition. The measured 2DEG density and mobility of the AlGaN/GaN heterointerface were 9.0 × 10^12^ cm^−2^ and 1500 cm^2^∙V^−1^∙s^−1^, respectively.

For device isolation, the active region of the device was defined by transformer-coupled plasma-reactive ion etching using a BCl_3_/Cl_2_ gas mixture. To protect the AlGaN surface from the high-temperature rapid thermal process (RTP) for ohmic contact formation, a 10-nm-thick Al_2_O_3_ dielectric layer was then deposited by atomic layer deposition. For the ohmic contact formation, the Al_2_O_3_ layer on the source/drain region was etched away and Si/Ti/Al/Ni/Au (1/25/160/40/100 nm) layers were deposited using an electron-beam evaporator. This was followed by a two-step RTP carried out first at a low temperature of 500 °C for 20 s and subsequently at a higher temperature of 800 °C for 30 s in nitrogen ambient. In addition, the Al_2_O_3_ layer on the gate region was etched away and Ni/Au was deposited as the gate metal. The wire bonding metal layer (Ti/Al) was deposited by electron-beam evaporation and patterned by a lift-off process. After the metal deposition, polyimide for the passivation layer was coated onto the whole wafer at 2500 rpm for 25 s and then cured for 50 min at 150 °C. After curing, the polyimide was patterned using a photolithographic process and then finally cured at 250 °C for 2 h to make a 2-μm-thick film. As shown in Figure 1b, the gate length and width of the fabricated sensor are 20 μm and 200 μm, respectively.

### 2.2. Measurement Procedure

**Figure 2:**
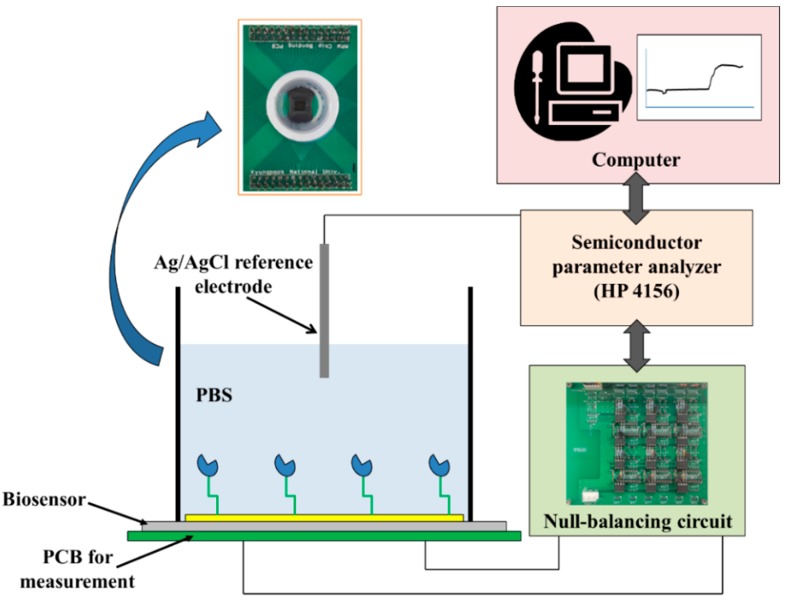
Measurement setup with the fabricated sensor chip packaged on a designed printed circuit board.

In order to evaluate the characteristics of the AlGaN/GaN HEMT-based biosensor, we prepared a measurement system. Figure 2 shows the measurement setup used. This system consists of a fabricated sensor embedded in a printed circuit board (PCB), a commercial Ag/AgCl reference electrode (RE-5B, BASi), a semiconductor parameter analyzer (4156, Agilent), and the null-balancing circuit. Epoxy and silicon rubber were coated on bonding wires for electrical isolation; only the Ni/Au gate area of the biosensor was exposed to the solution. The semiconductor parameter analyzer supplied all of the input biases, and detected the output signals. The reference electrode was immersed in PBS solution and can be used to apply the proper gate bias for the HEMT operation.

**Figure 3:**
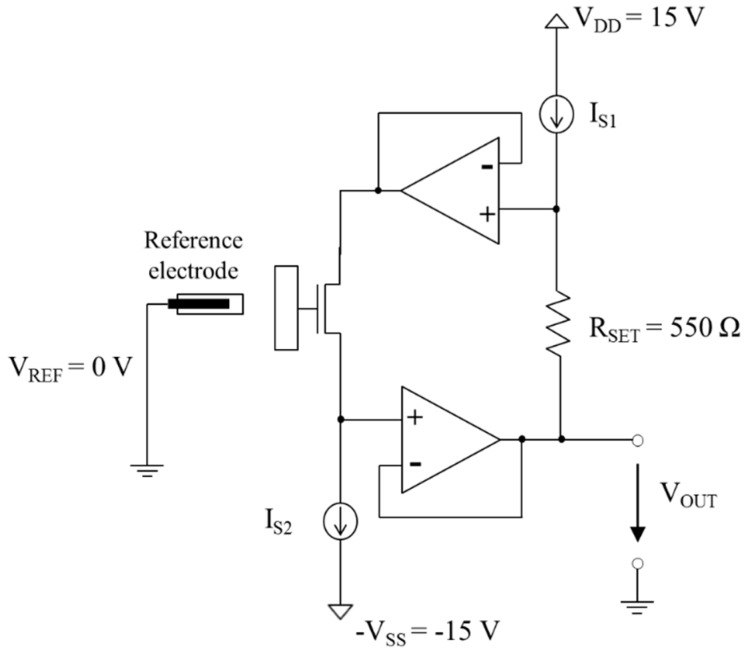
Schematic of the null-balancing measurement circuit.

Figure 3 shows a schematic of the null-balancing circuit, where the AlGaN/GaN HEMT-based biosensor is connected to an amplifier system. The drain current of the sensor is driven by the current source *I_S2_*, and the drain-source voltage *V_DS_* is fixed by the current source *I_S1_* and resistor *R_SET_*. Change in surface potential will lead to a change of the threshold voltage of the HEMT biosensor. HEMT biosensor operate in the linear region. When *V_REF_* = 0 V and *V_GS_* = *V_REF_* – *V_S_* = −*V_OUT_*, the expression for *V_OUT_* is derived as follows [14,15]:

$$V_{OUT}=\;-V_{th}-\left(I_{S2}/\beta ∙I_{S1}∙R_{SET}\right)-\left(I_{S1}∙R_{SET}/2\right)$$

When change in *β* (gain factor) are negligible and *R_SET_*, *I_S1_* and *I_S_*_2_ are constants, *V_OUT_* is proportional to a surface potential represented by the threshold voltage *V_th_* of the HEMT biosensor. Therefore, this circuit can measure the change in the potential of a FET surface (instead of the drain current) as a biomolecular immobilization occurs; it can also help in noise cancellation by using a differential structure [16,17].

In this experiment, we fabricated the sensing membrane by using the self-assembled monolayer (SAM) formation method on the Ni/Au gate region. This method has been used by a large number of researchers [18,19]. The CRP sensing membrane of the AlGaN/GaN HEMT-based biosensor was fabricated using the following chemicals: 11-mercaptoundecanoic acid (MUA, Sigma-Aldrich), N-hydroxysuccinimide (NHS, Sigma-Aldrich), N-(3-dimethylaminopropyl)-Nʹ-ethylcarbodiimide hydrochloride (EDC, Sigma-Aldrich), CRP from human plasma (Sigma-Aldrich), monoclonal anti-CRP antibody produced in mice (Sigma-Aldrich), and 1X PBS (GIBCO). The PBS solution, which has a pH of 7.4, was used as a sample solution throughout the experiment for the initial device stabilization and all subsequent measurements. Components of the buffer solution are 137 mmol/L NaCl, 2.7 mmol/L KCl, 10 mmol/L Na_2_HPO_4_ and 2 mmol/L KH_2_PO_4_. To start with, we injected 11-MUA solution (1 mmol/L in ethanol solution) on the surface of the Ni/Au gate region of the AlGaN/GaN HEMT-based biosensor for 24 h to form the SAM. Then, a solution containing NHS (50 mmol/L) and EDC (50 mmol/L) were used to create a stable amine-reactive product [18]. Next, the biosensor was dipped for 1 h in a 50 μg/mL CRP-antibody solution. Subsequently, Troponin T (1 μg/mL) was used to confirm the selectivity of the sensor. Troponins T protein is a component of the contractile apparatus of cardiomyocytes and is the preferred biochemical markers of myocardial necrosis in patients with suspected acute coronary syndromes [20]. The fabricated sensor was exposed to the Troponin T for 1 h. Finally, CRP antigen, the concentration of which varied from 0.01 ng/mL to 1000 ng/mL, was dropped onto the device for 3 h. Each concentration was repeated three times to obtain the standard deviation of the output voltage response. The AlGaN/GaN HEMT-based biosensor was rinsed in fresh PBS solution after the completion of each process.

XPS was used to evaluate the SAM adsorption on the gold surface. The spectra were acquired using a Quantera SXM system of PHI-ULVAC, equipped with a monochromatic Al Kα source and a hemispherical electron energy analyzer. Surface charging effects were avoided by using an electron flood gun. The binding energy shifts were calibrated by keeping the Au 4f peak at 84.0 eV. Two samples with Ni/Au deposited were prepared for the XPS experiment. The first sample was treated by dipping in 1 mmol/L 11-MUA solution for 24 h, whereas the reference sample was not treated. The XPS measurement provides information about the chemical structure within the films and can be used to study samples prepared in the ambient environment, without an additional in-situ treatment [21].

## 3. Results and Discussion

**Figure 4:**
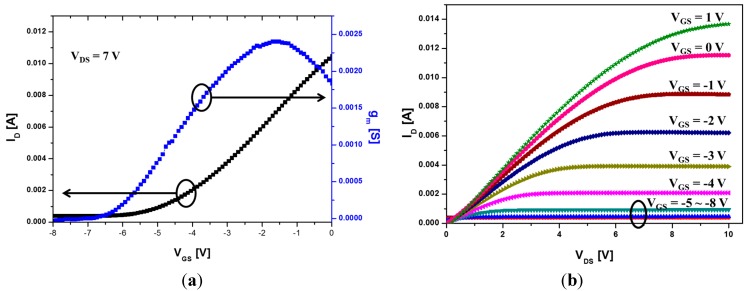
*I*-*V* characteristics of the AlGaN/GaN HEMT: (**a**) *I_D_*-*V_GS_*, *g_m_*-*V_GS_*; (**b**) *I_D_*-*V_DS_* characteristics.

The *I*-*V* characteristics of the AlGaN/GaN HEMT are shown in Figure 4. The fabricated device shows normally on operation with a threshold voltage (*V_th_* = −4.6 V), which is extrapolated from Figure 4a in the saturation region (*V_DS_* = 7 V). The device exhibits a maximum drain current of 13.6 mA at *V_GS_* = 1 V and *V_DS_* = 10 V, and a maximum transconductance of 2.4 mS at *V_GS_* = −1.5 V and *V_DS_* = 7 V.

**Figure 5:**
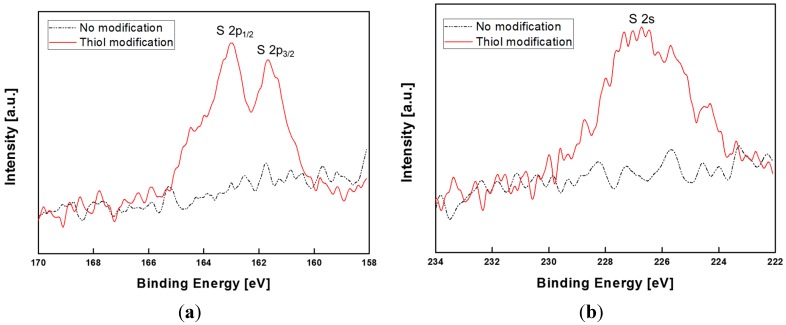
(**a**) S 2p; (**b**) S 2s high-resolution XPS spectra after SAM immobilization of the Au-deposited GaN sample.

XPS spectra were measured in order to examine the SAM immobilization on the Au surface. The SAM is adsorbed on the Ni/Au gate by reaction with thiol modification because of a strong interaction between Au and the thiol group. To confirm the modification was successful, XPS measurements were performed under the same conditions as the electrical measurements of the fabricated AlGaN/GaN HEMT-based biosensor. Figure 5 shows the measured high-resolution XPS spectra of S 2p and S 2s after SAM immobilization of the Au-deposited GaN sample. The S 2p featuring a binding energy of 161 eV and 163 eV indicates an Au thiolation sulfur species [22]. The S 2s peak at 227 eV was also observed for the SAM immobilized sample. Therefore, the results of the XPS measurements support the SAM formation on the sensing region of the fabricated sensor.

**Figure 6:**
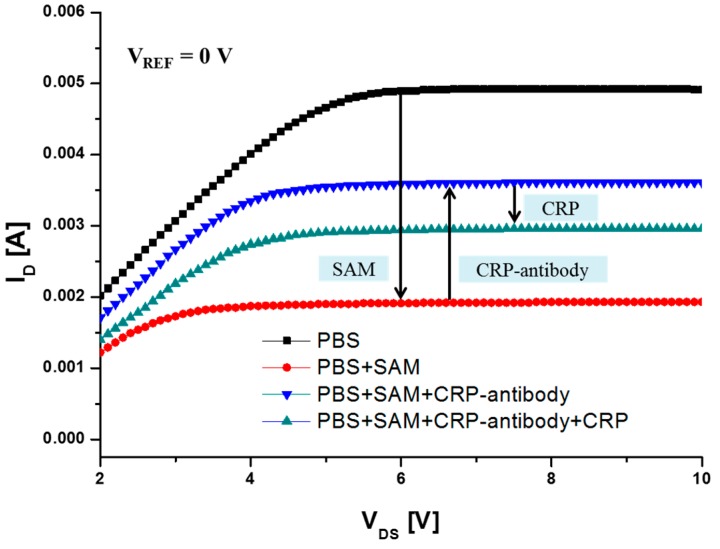
*I_D_*-*V_DS_* characteristics of the AlGaN/GaN HEMT-based biosensor as a function of interactions among SAM, CRP-antibody, and CRP (1000 ng/mL).

Figure 6 shows the *I_D_*-*V_DS_* characteristics of the AlGaN/GaN HEMT-based biosensor as a result of interaction among the SAM, CRP-antibody, and CRP. The drain current decreased after the SAM immobilization by 2.98 mA at *V_DS_* = 8 V. In that case, the negative surface potential on the gate was introduced because of the net negative charge of the reactive thiol head group of the SAM. After the formation of the SAM layer, the device was exposed to CRP-antibody. The positive charge of the CRP-antibody amine group, which is closer to the gate surface, attracts electrons in the HEMT 2DEG channel [13]. Therefore, the drain current of the sensor increased by 1.68 mA when CRP-antibody bound to the SAM. Finally, CRP was injected to the device, binding specifically with the previously immobilized CRP-antibody molecules. The antibody-antigen binding changes the net charge of the gate region of the sensor. As a result, the negative charge of the CRP decreases the drain of the sensor.

**Figure 7:**
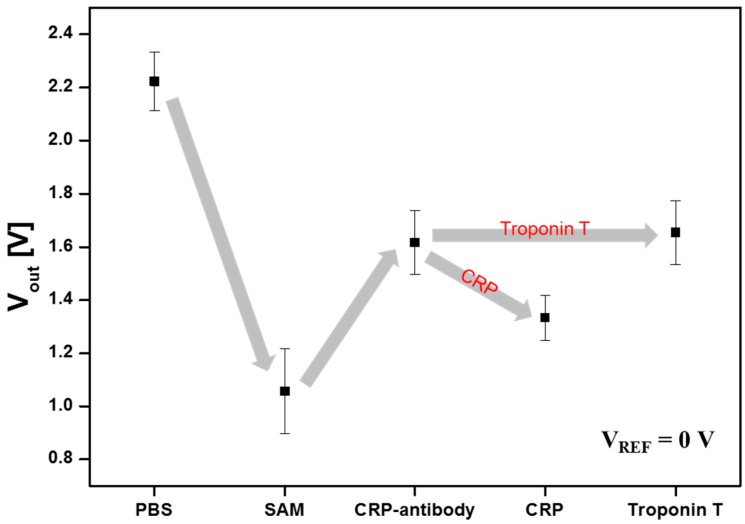
Output voltage characteristics of the AlGaN/GaN HEMT-based biosensor with null-balancing circuit applied (1000 ng/mL CRP and 1000 ng/mL Troponin T).

Furthermore, the output voltage variation of the AlGaN/GaN HEMT-based biosensor with null-balancing circuit applied can also be used to detect the reaction of CRP-antibody/CRP protein complexes by results for output voltage characteristics of the sensor as shown in Figure 7. The presence of the transport of associated charge and a change of the net capacitance by an accumulation of an organic layer will also affect channel conductance equivalent to a change in gate bias [23]. These results are shown as a same tendency with the *I_D_*-*V_DS_* characteristics of Figure 6. In order to test the selectivity of the sensor, Troponin T protein was injected with a concentration of 1 μg/mL to the sensor. According to the results, drain current was not changed for Troponin T with the CRP-antibody, whereas about 0.3 V of output voltage difference was measured after CRP injection on CRP-antibody. These data prove that the target CRP reacted only with CRP-antibody. Thus, it is concluded that the drain current variation in the biosensor occurred because of the specific binding of CRP-antibody and CRP, and not by non-specific binding to other biomolecules.

**Figure 8:**
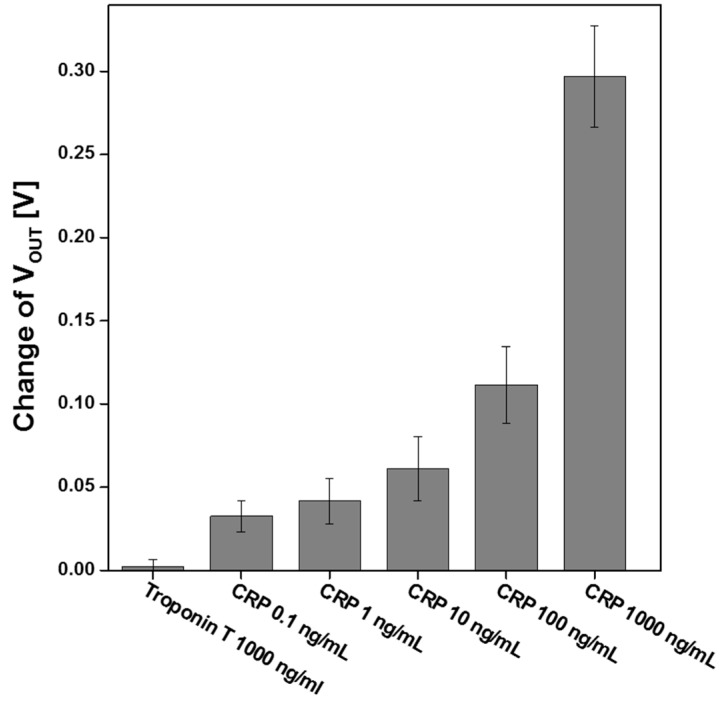
Variation of *V_OUT_* with concentration of CRP.

As shown in Figure 8; the output voltage change in the biosensor as a function of CRP concentration was measured to quantify the limit of detection (LOD) for CRP. The device was exposed from 0.01 ng/mL to 1000 ng/mL; and each concentration was repeated three times to obtain the standard deviation of output voltage difference for each concentration. Only a slight change was observed when from 0.01 ng/mL to 1 ng/mL CRP was injected; therefore; the LOD of this device is 10 ng/mL CRP in PBS solution; and the output voltage change is nonlinearly proportional to CRP concentration. Commercial ELISA kits have a LOD of 0.1 ng/mL and a detection range of 0.1 ng/mL to 5 ng/mL [18]. The proposed sensor has a lower LOD than commercial ELISA kits but a wider detection range than commercial ELISA kits. The AlGaN/GaN HEMT-based biosensor with null-balancing circuit could detect CRP molecules at concentrations as low as 10 ng/mL; because AlGaN/GaN HEMT has a high electron mobility induced by piezoelectric polarization. In addition; the strong polarization field in AlGaN/GaN material responds strongly to the biomolecule charges on the gate surface [13]. Moreover; the LOD of the device may be improved by using a differential structure for noise cancellation [16,17].

## 4. Conclusions

In this study, we demonstrated a highly sensitive AlGaN/GaN HEMT-based biosensor with a null-balancing circuit for the detection of CRP. The electrical characteristics of the fabricated devices were measured and investigated. A null-balancing circuit was used to measure the output voltage of the biosensor directly, instead of measuring the drain current of the device. The immobilization of SAM on the gate surface was verified by XPS measurement.

By modifying the Ni/Au gate region with CRP-antibody immobilization, the target CRP can be selectively detected through the immune reaction. After the reaction among the SAM, CRP-antibody, and CRP, the variation of the drain current and the output voltage revealed a change of the surface charge. In addition, experimental results show that the AlGaN/GaN HEMT-based biosensor exhibits good selectivity over a wide sensing range of 10 ng/mL to 1000 ng/mL.

From the above results, we conclude that the proposed biosensor might be successfully applied in a system for the diagnosis of cardiovascular disease.
